# The model of erosive hand osteoarthritis

**DOI:** 10.1186/ar3715

**Published:** 2012-03-08

**Authors:** Margreet Kloppenburg

**Affiliations:** 1 Department of Rheumatology, Leiden University Medical Center, Leiden, The Netherlands

## 

Hand osteoarthritis (OA) is not one disorder, but a heterogeneous group of disorders. Erosive HO (EOA) is a radiographic subset of hand OA and defined on the basis of radiographic central erosions and collapse of the subchondral bone plate. At the moment it is unknown whether EOA comprises a separate disease entity with specific risk factors and pathogenesis or a more severe stage of hand OA.

EOA is a rather uncommon subset of OA. In the general population a prevalence of EOA in the interphalangeal joints (IPJs) of approximately 3% was estimated in adults aged 55 years and over. In the population with hand pain or with symptomatic hand OA the prevalence estimates were around 7% and 10%, respectively. However in a population with symptomatic hand OA from secondary care, the prevalence of EOA raised to 25%.

The presence or absence of specific risk factors for EOA could learn whether EOA is a separate disease entity or a severe stage of hand OA. The first step in this research deals with heritable factors. Several studies suggest that EOA is heritable. Within patients with hand OA, several genetic factors are demonstrated to be associated with EOA when compared to non-erosive hand OA, but further replication of found results is needed.

EOA is a highly clinical relevant subset, since EOA leads to a higher clinical burden and worse outcome than non-erosive hand OA. Patients with EOA report more pain and functional limitations, worse hand mobility, less satisfaction with hand function and aesthetics than those with non-erosive hand OA. However patients with EOA have also more nodes, which were also found to be a determinant of clinical outcome. Taking in account nodes, only hand mobility and patient satisfaction remained different between the groups.

In the pathophysiology of EOA inflammation could be important. Already since 1972 it is noted that inflammation is involved in EOA, as also witnessed by histology of synovial biopsies of erosive DIPJs and PIPJs in an inflammatory stage, showing intense proliferative synovitis indistuinguishable from rheumatoid arthritis. Recently the presence and role of inflammation is further investigated by ultrasound and MRI studies. Moreover, ultrasound studies showed that not only in erosive but also in non-erosive joints in EOA more power Doppler signal and effusion is seen in comparison to joints in non-erosive hand OA, suggesting an underlying cause for erosive evolution.

Another important observation is that erosions are more frequently present in hand OA than based on radiography when using ultrasound. The meaning of erosions only detected on MRI and ultrasound has to be elucidated.

In conclusion, EOA is a highly clinical relevant subset of hand OA. Whether it is a separate disease entity is at the moment unknown, but needs further study. Inflammation seems of importance, already in the pre-erosive stages. Further research in this hand OA subset could lead to new treatments for patients with EOA.

